# Supplementary material for: Sexual and reproductive health service utilization among adolescent girls in Kenya: A cross-sectional analysis
Source: PLOS Glob Public Health. 2023 Feb 22;3(2):e0001508. doi: 10.1371/journal.pgph.0001508 (PMC10021741; doi:10.1371/journal.pgph.0001508)
Supplement: Attachment — Submitted filename: Response_Reviewers.docx [file pgph.0001508.s001.docx]

***PLOS GLOBAL PUBLIC HEALTH***

November 21^st^, 2022

**Re:** PGPH-D-22-01261
Sexual and reproductive health service utilization among adolescent girls in Kenya: a cross-sectional analysis

Dear Anil Gumber,

We are writing this letter to address the reviewer comments concerning the submitted manuscript: ‘*Sexual and reproductive health service utilization among adolescent girls in Kenya: a cross-sectional analysis*’*.* We would like to thank the Editor and Reviewers for their valuable comments. This letter addresses these comments numerically point by point to accompany the attached revised manuscript, which highlights changes highlighted in yellow. The comments of the reviewers are italicized followed by our corresponding responses.

**Journal Requirements:**

*1. Please amend your detailed Financial Disclosure statement. This is published with the article. It must therefore be completed in full sentences and contain the exact wording you wish to be published.*

*a. Please clarify all sources of funding (financial or material support) for your study. List the grants (with grant number) or organizations (with url) that supported your study, including funding received from your institution. 
b. State the initials, alongside each funding source, of each author to receive each grant.
c. State what role the funders took in the study. If the funders had no role in your study, please state: “The funders had no role in study design, data collection and analysis, decision to publish, or preparation of the manuscript.”
d. If any authors received a salary from any of your funders, please state which authors and which funders.
If you did not receive any funding for this study, please simply state: “The authors received no specific funding for this work.”*

We have provided the funding information on page 17 of the manuscript. We have updated it with the text (pg. 25 lines 423-428) below to reflect all the required statements:

The In Their Hands (ITH) project evaluation was funded by Children’s Investment Fund Foundation (CIFF) through a grant to the African Population and Health Research Center (APHRC), Grant Number - R-1710-02085. APHRC, represented by two of the co-authors - YDW and CO - led the evaluation study. The funders had no role in study design, data collection and analysis, decision to publish, or preparation of the manuscript. The authors received no specific funding for this publication.

*3. In the online submission form, you indicated that "Data is available upon reasonable request". All PLOS journals now require all data underlying the findings described in their manuscript to be freely available to other researchers, either 1. In a public repository, 2. Within the manuscript itself, or 3. Uploaded as supplementary information.*

We have now amended this statement. The data is available in a public repository at APHRC. The data from this analysis can be accessed by submitting a data request in the APHRC Micro-data portal [https://aphrc.org/microdata-portal](https://urldefense.proofpoint.com/v2/url?u=https-3A__aphrc.org_microdata-2Dportal&d=DwMFaQ&c=shNJtf5dKgNcPZ6Yh64b-ALLUrcfR-4CCQkZVKC8w3o&r=uXl-2pd4SzSx5VTP0cHYRVoK8GU6--6MkPNA-u_45fg&m=s3dvXfwx9HaZPFZSopu1xAs4WSToOxI7QqMxp1irmSiMuif2dtGo830gJscbDdgy&s=RykO7yqvikLV6YeJleTOpsdyDbBLLimWcJShWnw9wcE&e=)

**Editorial Comments to the authors:**

*An important subject has been covered by the authors. However there are some methodological issues and analysis need further strengthening. The study has tried to identify access and utilisation rates across two regions; instead they should have focus on the determinants and association in rural and urban areas separately. We understand the access to healthcare facilities are much better in urban than in rural areas; however, a surprise findings/differences do appear in rural and urban areas but the utilisation of sexual and reproductive services was much greater in rural than urban adolescent girls. Certain statistics are provided for 10-19 age groups whereas the analysis focused on 15-19 age group; one needs to re-do those statistics relevant for 15-19 age group. Several tables no statistical significance tests were attempted. Authors need to present all tables/analysis separately for rural and urban areas. The discussion needs to be improved in the context of rural-urban differences in sexual health seeking behaviour.*

We would like to thank the Editor for these comments and suggestions. We would like to clarify that this analysis examined the association between adolescent sexual and reproductive service utilization in the past 12 months and structural, health facility, community, interpersonal, and individual level factors across the social-ecological model as outlined in the introduction on page 5 lines 123-125. We sought to identify key barriers and facilitators at each of these levels that may be targeted to improve access to and utilization of SRH services for adolescents in this context.

Of note, this analysis only contains participants, who are adolescent girls aged 15 to 19 years, and not participants aged 10-19 years as stated on page 6 lines 139 and 150-151.

Further, we found no association in our unadjusted analysis between geographic location (urban vs. rural) and sexual and reproductive health service utilization across adolescent girls aged 15-19 years in Kenya (OR: 0.85 95% CI: 0.70-1.03) as shown in Table 2.

Additionally, unadjusted and adjusted odds ratios are provided across Tables 2-5 as appropriate. We conducted unadjusted logistic regression analyses to examine the relationship between each variable and SRH service utilization. Variables were selected for multiple logistic regression models based on bivariate analyses significance (*p* < .05). We fit four separate adjusted multiple logistical regression models for each of structural, health facility, community/interpersonal, and individual level factors. All models were adjusted for county, age, relationship status, orphan status, and religion. All analyses were conducted in RStudio Version 1.4.1106.

Given that our analysis did not focus on rural-urban differences as this factor was not-significant in the analysis as demonstrated in Table 2 (OR: 0.85, 95% CI: 0.70-1.03), we have not modified our discussion at this time to reflect this domain as it did not play a role in sexual and reproductive health service utilization for adolescent girls in this analysis.

**Reviewer 1**

*Lines 182-184: Say more about this for adolescents and SRH- there are issues with these gatekeepers. How did this affect respondents especially those below 18?*

Thank you for this comment. We have added additional text on lines 155-175 to elaborate on sampling and recruitment, and response rate with the following text:

The study team conducted community mobilization and sensitization in the study sites before any household listing and data collection activities. Supervisors and team leaders led community engagement at the village level and obtained approvals from community leadership to carry out the survey in the site. Village elders and local guides supported the team when entering communities and locating households. At the county and sub-county levels, the research team organized meetings with county commissioners and county health and presented study approvals and permits obtained from AMREF ESRC (AMREF-ESRC P499/2018) and NACOSTI respectively.

The response rate for this study was 97%. Of the 1897 households with eligible respondents, 1840 adolescent girls were successfully recruited and participated. Fifty-seven adolescent girls did not participate due to a lack of parental consent, unavailability, or refusal to participate. This is outlined in the Methods sub-section Sample size, sampling, and recruitment on lines 164-168. We believe that by working with the community health volunteers and local Chiefs and Village Elders to sensitize the community and parents about the study, we were able to gain community entry, mobilize study participants and have a very high response rate.

*Lines 182-184 - did you also involve schools?*

Schools were not involved in the process to recruit and mobilize study participants or in any data collection activities. The survey was community based.

*Lines 243-244 what type of SRH services for those In school and for those out of school. For the in school, did you consider seeking services during weekends or during school breaks?*

As this analysis used baseline data from Homa Bay and Narok counties, SRH service utilization, the primary outcome of interest, participants were asked if in the past 12 months they had visited a health facility for sexual and reproductive health services. We did not specifically inquire whether they sought services on weekends or during school breaks. The way the question was worded ‘In the last twelve months, have you ever visited a health facility to receive sexual and reproductive health services’. This would be inclusive of evenings, weekends, and school closures.

*Lines 245 – 246 permissions from who? Was getting permission from parent to seek SRH services included in this? + comment in table - did you compare this for those in school and those out of school? given the issues of stigma around SRHR would be good to contextualize this in the methods section*

The questionnaire did not specify who permission was sought from if it was an issue, so parental or spousal/partner permission was not included specifically in this. The survey question was many different factors can prevent girls from getting medical advice or treatment for themselves. When you want to get advice or services on sexual and reproductive health, is each of the following a big problem or not. Getting permission to go (yes/no). We did not compare across in and out-of-school participants if getting permission to go as it was part of our adjusted model. We included education as a structural factor in Table 2. As you can see in Table 2, when adjusting for age, relationship status, orphan status, and religion, across structural factors, county, school attendance, and getting permission to go all remained significantly associated with SRH utilization. However, you can see that the relationship between getting permission to go (no) and school attendance is attenuated when adjusting for these variables.

*Table2 - did you check distances per county? Narok would most likely have longer distances to health facility compared to Homabay*

Thank you for this comment. Unfortunately, distance to the facility from the participants location was not measured. This response ‘distance to the health facility is a problem’ was part of the list of factors that may prevent girls from getting medical advice or treatment for themselves as outlined above with a yes/no response. As this factor was not significant in the analysis, it appears that adolescent girls did not think that distance was a factor preventing SRH utilization. We did not analyze this factor by county.

*Table 3 - was this just a provider or a provider who is friendly and supportive to the adolescents*

As you can see in table 3, we included separate variables for concern that the provider may not be adolescent-friendly and concern that there may be no provider (e.g. no provider will be present at the health facility).

*Line 291 – 293 out of school??*

This analysis was not stratified by school status (in versus out of school). Table 5 looked at individual level factors associated with SRH utilization. First, we conducted unadjusted logistic regression to look at the association between ‘engaging in work in work for money’ and ‘SRH utilization’ which was significant (OR: 2.00 95% CI: 1.6-2.52). Individual level factors that were significant were then run in an adjusted model for the individual level factors controlling for county, age, relationship status, orphan status, and religion.

*Lines 295 – 297 how did this look like per county and for in and out of school*

The analysis adjusted for county, age, relationship status, orphan status, and religion, while holding all other co-variates constant in the adjusted individual level model. We did not stratify our analyses by in and out-of-school status or by county as we were attempting to isolate factors across the structural, health facility, community, interpersonal, and individual levels that are associated with SRH utilization. As you can see in Table 1, county and school attendance were significant. We adjusted for county across all models as a potential confounder.

**Reviewer 2**

Reviewer #2: This paper presents the results of a cross sectional survey investigating associations between adolescents’ sexual and reproductive health service utilization and structural, health facility, community, interpersonal, and individual level factors in Kenya. In general it is robust, thorough, has a strong methodology and interesting results which are well interpreted.

The below comments are suggested to tighten up the paper, with a few considerations to add to the discussion / limitations.

*P6 Study setting highest adolescent pregnancy .. please add age cut off – is it 15-18y,10-18y, 15-19y, 10-19y*

We have now included that this was among adolescents aged 15-19 years and the DHS reference.

*P7 Study participants, sampling – please clarify for sub-county, wards, and villages, how you sampled. Similarly, how was random sampling done of the adolescent girls per household*

Lines 155 – 175 outline the sampling strategy by sub-counties, we clarify below and have added additional text as necessary to provide more detail.

Within the three sub-counties identified, the ITH evaluation selected wards that were located within the catchment areas of health facilities identified for the intervention. This is because the intervention involved both demand creation (mobilization of adolescents in the communities for the program) and health facility strengthening by training providers and subsidizing services. All villages located within the catchment area (within 10 km radius from the health facility) were included in the survey. Household listing was done in all the villages to identify households with eligible adolescents. Once households with eligible adolescents are identified, household roster (listing of all household members) was done collecting demographic information of each household member. In cases where there were more than on eligible adolescent, one adolescent was randomly selected by the SurveyCTO program. SurveyCTO has the ability to automatically randomize the selection (using the Random function or a form of Kish Grid) if programmed without any input from the field interviewer.

The sampling and recruitment section on lines 154-175 now reads:

*Sample size, sampling, and recruitment: Using Cochran’s formula, a sample size of 1885 was calculated to detect differences in participants’ intention to use contraception in the future between baseline and endline. The study team conducted community mobilization and sensitization in the study sites before any household listing and data collection activities. Supervisors and team leaders led community engagement at the village level and obtained approvals from community leadership to carry out the survey in the site. Village elders and local guides supported the team when entering communities and locating households. At the county and sub-county levels, the research team organized meetings with county commissioners and county health. Sampling lists were then created based on a process that identified all eligible adolescent girls residing in households in all villages within health facility catchment areas (within a 10km radius from the health facility) affiliated with the intervention. Within each county, 3 sub-counties were sampled, which included West Kasipul, Ndhiwa and Kasipul in Homa Bay, and Narok Town, Narok South and Narok East in Narok county. In each sub-county, three wards were selected, and villages were selected within the wards. In total, 22 villages in Narok and 24 villages in Homa Bay were sampled. In each of the villages, household sampling lists were generated where adolescent girls resided. A total of 1897 households with eligible respondents were identified. One adolescent girl was randomly selected per household to participate in the survey by the SurveyCTO program. The response rate for participation was 97%. In total, 1840 adolescent girls were successfully recruited and participated in the cross-sectional survey; 57 (3%) adolescent girls selected did not participate due to lack of parental consent, unavailability, or refusal to participate.*

P7 What was the time lag between the generation of the sampling frame for adolescent girls aged 15-19y and the adolescent survey? (i.e. some of girls reached 20y?

Data collection was completed immediately after the generation of the sampling frame. The survey questionnaire also included questions on date of birth and age that were used to exclude adolescents that reached 20 years.

*P7 Consider noting the percent refusals e.g., 57 (3%) were refusals or unavailable.*

We have now included the percentage.

P7-8 No mention is made of consenting of full orphans who were under age 18 – did they all live with guardians and have guardian consent or were some emancipated minors?

We have included a statement regarding the consent of emancipated minors on line 177 with the following text: Married and other emancipated minors and those aged 18 years or older provided written informed consent to participate. For minors living with their guardians, informed consent was obtained from the guardian and assent from the minor.

*P8 In line 185 note typo ‘each selected household’ (no s)*

We have removed this typo.

*P8 Kiswahili as local language is a surprise – in Homa Bay Luo the local language and Table 1 indicates 58% of the survey population were Luo – were there any issues using Kiswahili? Also, were there are any illiterate out-of-school girls who required face to face interview instead of completing electronic tablet for the survey? In rereading, I could not see if the survey was self-completed or by interviewer (maybe I missed).*

In both Homa Bay and Narok counties, Kiswahili was the main language used for interviews although English was also used for those who were fluent and preferred English. There was no major issue using Kiswahili in Homa Bay. It was rather in Narok that a few of the sampled adolescents were not very fluent and required interviewers help. In both counties, we used interviewers who can speak the local language although they were advised not to try to translate the questions. The survey was programmed on a tablet and administered by a trained interviewer face to face. It was not self-administered.

*P9 – any attempt to differentiate sexual activity in past 12 months between ‘willing’ and ‘unwilling’ (forced, tricked, etc)*

This survey did not distinguish between consensual and forced sexual activity in the past 12 months.

*P9 – shame there was nothing on household economics (as a household survey, and nested in larger study, was there a parallel socio-economic status indicator at household level – one would imagine relative poverty would be an important measure to include in analyses.*

Thank you for this thoughtful comment. Unfortunately, household level socio-economic information was not collected as part of this evaluation and no parallel socio-economic indicator is available at the household level in the data set. If participants reported, they were engaged in work or out-of-school this may be an indicator of poverty.

Results
*P9-10/Table 1 – orphan status seems to include unknown as well as both parents died; who are the ‘unknowns’ and what proportion are they of the 150 ‘double orphans’ category - wondering if these have a diluting effect on any analyses?*

Only 2 participants responded, ‘don’t know’. For these two participants, we presumed they were separated from both of their parents and did not know their vital status, effectively leaving them as double orphans, and therefore grouped them in with double orphans.

*P11 Authors noted that about half of participants reported their SRH service was receiving an HIV test during their visits to a facility, followed by general care (29%), contraception (27%), and antenatal care (24%). Have the authors explored whether there were significant differences within their model e.g. at interpersonal, personal level for these key services – would it be worth having as a supplementary table if there were differences ?*

We did not explore across each model the association between structural, facility, community, interpersonal, and individual level factors and by type of services sought during the visits to the health facility. It is possible that different factors are associated with utilizing different types of SRH services. The aim of this paper is to examine SRH service utilization broadly, given that SRH service utilization is low among adolescent girls and young people, to identify factors that can be targeted to improve SRH utilization in this context. We fear that this will create an abundance of tables that are beyond the scope of the manuscript.

*Adjusted models by relationship status to control for exposure to SRH harms, did authors also look at reported sex / no sex, with the latter ~40% of the population (n=730)?*

We did not adjust for sexual activity across the models, as young people may seek sexual and reproductive health services, and in an ideal world, should seek sexual and reproductive health services prior to sexual activity to be advised and counseled on family planning, HIV testing and counseling, and other sexual and reproductive health issues.

*Discussion
Well written and thorough. Authors may wish to add that they were unable to include socio-economic status of their household to the limitations paragraph.*

We have now added this as a limitation in our discussion on lines 388-389 with the following text: Our study did not collect data on household level socio-economic status, which may be an important factor associated with adolescent girls’ SRH utilization.

We thank the reviewers for their feedback on our study.

We hope we have adequately addressed the reviewer’s comments regarding this manuscript. We welcome additional comments and criticism that will assist us in disseminating this important information.

Sincerely,

The Authors
